# Smoking, *APOE*, and Dementia: From Effect Modification to Mechanism and Prevention

**DOI:** 10.1016/j.bpsgos.2026.100829

**Published:** 2026-10-28

**Authors:** Nasri G. Fatih, Anya Topiwala

**Affiliations:** Big Data Institute, Nuffield Department of Population Health, University of Oxford, Oxford, United Kingdom

In a recent article published in *Biological Psychiatry: Global Open Science*, Cong *et al.* report evidence that the association between smoking and incident dementia differs by *APOE* ε4 status, with stronger relative associations among ε4 noncarriers (1). In the UK Biobank (UKB), they followed 173,366 participants without dementia at baseline for a median of 13 years and, using two composite indices derived from self-reported smoking history, identified significant multiplicative interactions between smoking and *APOE* ε4 genotype for all-cause dementia. This pattern was supported by a meta-analysis of longitudinal studies. Plasma proteomics implicated inflammatory and angiogenesis-related pathways in the associations between smoking and dementia and highlighted digestive system and phospholipid pathways as potential contributors to its modification by *APOE* ε4 status. The authors propose that these findings could inform dementia risk stratification and prevention. However, the interaction also raises more fundamental questions: what does it represent biologically, which smoking-related pathways or dementia processes might it account for, and, crucially, does an interaction on the relative risk scale translate into meaningful differences in absolute dementia risk or the expected benefit of smoking cessation?

The interaction reported by Cong *et al.* is statistically compelling; however, its interpretation requires nuance. The authors observed multiplicative effect modification between smoking and *APOE* ε4, but no additive interaction was observed. As Hunter emphasized (2), the presence and magnitude of gene–environment interaction depend on the statistical scale used. This is particularly relevant because *APOE* ε4 confers substantially higher underlying risk of Alzheimer’s disease, especially among ε4 homozygotes (3). The same absolute increase in dementia risk associated with smoking could therefore translate into a smaller relative effect among ε4 carriers. A lower hazard ratio in carriers should not, by itself, be interpreted as evidence that smoking has less absolute impact in this group. Cong *et al.* themselves acknowledge this possibility (1) (Figure 1).

**Figure 1:**
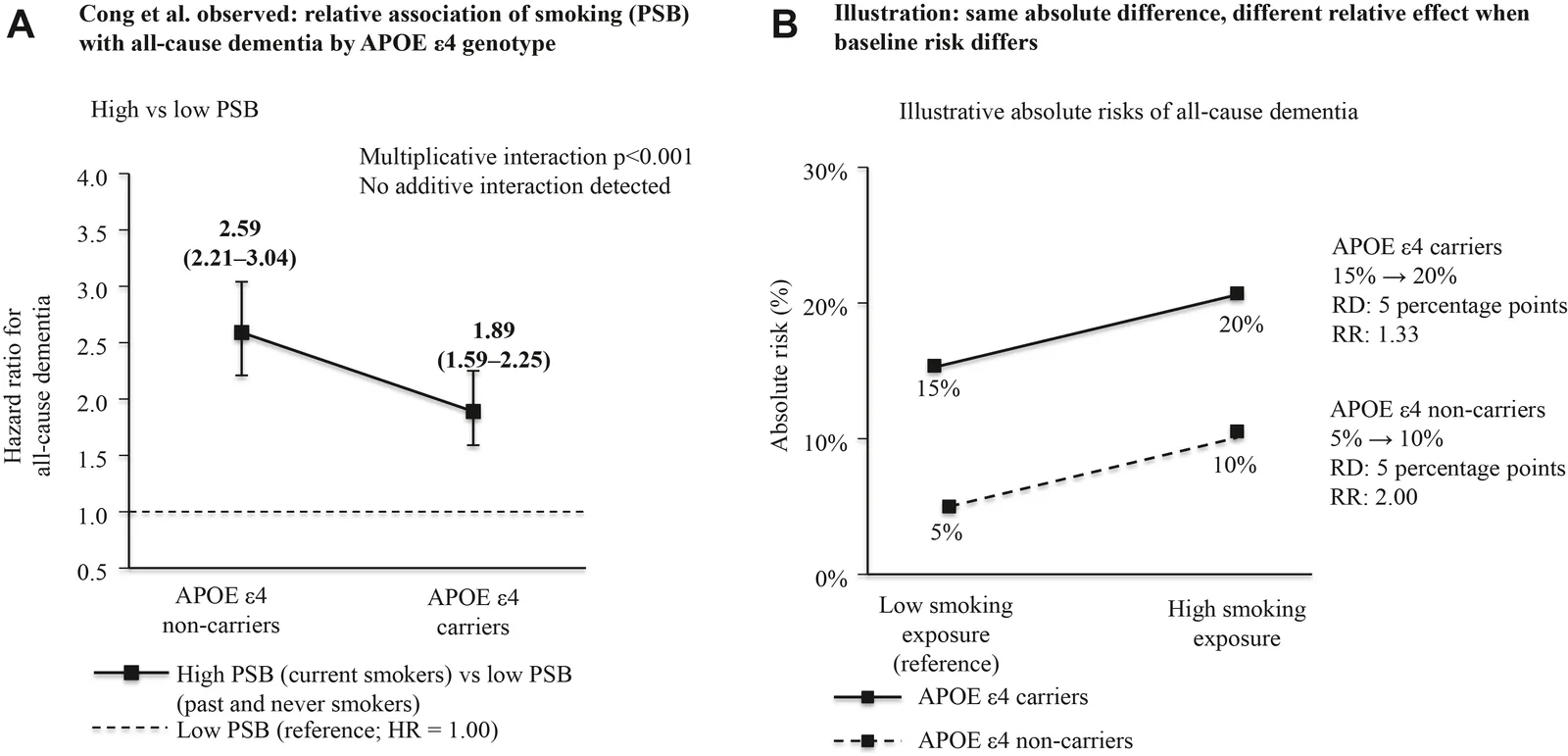
Smoking, *APOE* ε4 genotype, and risk of all-cause dementia: study findings and epidemiological illustration. Panel **(A)** shows adjusted hazard ratios (95% CI) for high versus low panoramic smoking burden (PSB) among *APOE* ε4 noncarriers and carriers reported by Cong *et al.* (1). Panel **(B)** uses hypothetical absolute risks to illustrate how identical risk differences (RDs) can correspond to different relative effects when baseline risk differs; values in (**B)** are not estimates from Cong *et al.* (1)*.* RR, risk ratio.

Selection into UKB also warrants consideration. Participants are healthier than the general population, with lower rates of smoking, morbidity, and mortality (4). Smokers who survive and are sufficiently healthy to enter the cohort may therefore represent a selected subset of smokers in the source population. Competing-risk analyses appropriately account for deaths after recruitment but cannot address selection or selective survival before enrollment. Whether this differs sufficiently by *APOE* genotype to influence the observed interaction remains uncertain, but importantly, the absence of an association between *APOE* ε4 and death during follow-up cannot exclude preenrollment selection.

Exposure measurement introduces further uncertainty. Cong *et al.* improve on simple smoking status by constructing two composite indices: a poly smoking burden incorporating smoking initiation, age at initiation, heaviness, and current/former status, while a smoking history score incorporating smoking status and pack-years (1). However, both rely on smoking information collected at baseline, and cannot capture changes over 13 years of follow-up or, importantly, time since cessation (1). Moreover, their construction was partly informed by their observed associations with dementia within UKB (1), and the high poly smoking burden category consists entirely of current smokers. Consequently, it is difficult to isolate whether the interaction is driven principally by current smoking, cumulative exposure, recency of exposure, or another component of smoking history. External validation of these indices would be valuable; the accompanying meta-analysis supports the broader smoking-*APOE* interaction, but does not directly validate either measure and is not wholly independent because one study also used UKB. Objective biomarkers such as cotinine could complement self-reported smoking for classifying current exposure, although they would not resolve uncertainty around cumulative exposure or changes in smoking behavior over follow-up (5).

Finally, the estimand itself warrants consideration. The models adjust for several vascular and metabolic conditions, including hypertension, hyperlipidemia, and stroke (1), some of which may mediate the effects of smoking on dementia. Such adjustment may be appropriate for prediction or for estimating a conditional association, but it complicates the interpretation of the interaction as a modification of the total effect of smoking.

These considerations do not weaken the evidence that the smoking–dementia association varies by *APOE* genotype; rather, they sharpen its interpretation. The study demonstrates statistical effect modification but is less able to identify the causal contrast responsible for it.

If the observed effect modification reflects genuine biology, the next question is how smoking contributes to dementia in different genetic contexts. Biological interaction is plausible: smoking and *APOE* both influence inflammatory, vascular, and lipid-related processes relevant to dementia, while smoking-related oxidative stress provides another potential mechanism (6,7). However, plausibility is not specificity. Cigarette smoke is a complex exposure (2), and *APOE* has pleiotropic effects across lipid transport, amyloid biology, and cerebrovascular function (7); their interaction does not therefore identify which pathway is responsible.

Greater biological specificity may come from examining dementia pathology. *APOE* ε4 is strongly associated with Alzheimer-type pathology (7), whereas an important component of smoking-related dementia risk may operate through vascular injury (6). Differing contributions of Alzheimer, vascular, and mixed pathology to all-cause dementia could, in principle, generate heterogeneity in the observed smoking association across *APOE* strata without requiring a single *APOE*-dependent mechanism. Notably, the accompanying meta-analysis did not detect a statistically significant smoking × *APOE* interaction for Alzheimer’s disease specifically, although the smaller subtype-specific evidence base limits inference from this null result. Subtype-specific and ideally biomarker defined outcomes in larger datasets could therefore be particularly informative.

The proteomic analyses offer intriguing mechanistic leads, but they do not yet resolve this question. Twelve proteins showed significant mediation effects among *APOE* ε4 noncarriers, compared to none among carriers (1). However, demonstrating mediation of the smoking–dementia association within genotype strata is not equivalent to demonstrating that those proteins mediate the smoking × *APOE* interaction itself. Differences in statistical significance between strata do not constitute a formal test that the indirect effects themselves differ by *APOE* status. Temporality also warrants caution: smoking exposure was characterized from baseline smoking history and status, while the mediation analyses do not incorporate longitudinal changes in either smoking behavior or the proteome. Repeated exposure and proteomic measurements, coupled with formal tests of *APOE*-dependent mediation, would provide stronger evidence that these pathways explain the interaction rather than merely accompany it. Ultimately, the biologically informative question is not simply whether smoking interacts with *APOE*, but which aspect of smoking exposure, which dementia process, and which molecular pathway give rise to that interaction.

The public health interpretation requires particular care. A smaller relative smoking–dementia association among *APOE* ε4 carriers should not be interpreted, or communicated, as evidence that smoking matters less for those at greater genetic risk. The headline hazard ratios describe differences on a relative scale and do not directly quantify the absolute difference in dementia risk associated with smoking within each genotype group. Likewise, the absence of an additive interaction does not establish that the absolute risk increases from smoking, or the benefit achievable through cessation, is identical across genotypes. Population-attributable risk estimates answer another question, reflecting both the strength of association and the exposure prevalence. Relative interaction, additive interaction, population-attributable risk, and individual preventive benefit are therefore not interchangeable.

For prevention, genotype-specific absolute risks under continued smoking versus cessation would be more directly informative: does the absolute reduction in dementia risk associated with cessation differ meaningfully between ε4 carriers and noncarriers? Similarly, the presence of statistically significant interactions within male and late-life strata, but not in female or mid-life strata, does not itself establish that effect modification differs by sex or age; that requires formal tests of between-group heterogeneity.

Until such differences are demonstrated, *APOE* status provides little reason to alter the fundamental preventive public health message. The 2024 Lancet Commission on dementia prevention identifies smoking as a major modifiable risk factor and emphasizes that “Risk is modifiable irrespective of *APOE* genetic status” (8). Smoking cessation should therefore remain a universal recommendation rather than one conditioned on *APOE* genotype. The immediate value of the interaction may lie less in identifying who should stop smoking than in improving our understanding of why smoking-related dementia risk may differ across biological contexts.

Cong *et al.* provide evidence that the relative association between smoking and dementia differs by *APOE* ε4 status, while their proteomic analyses offer plausible mechanistic hypotheses for this heterogeneity. The challenge now is to move from statistical interaction to biological explanation and to establish whether that understanding has implications for prevention. This will require replication in independent populations, better characterization of the smoking exposures driving the interaction, and investigation within specific, ideally biomarker-defined, dementia pathologies. It will also require evidence that the proposed molecular pathways mediate the interaction itself, rather than the smoking–dementia association more generally, and determining whether genotype meaningfully alters the absolute benefit associated with smoking cessation. Until then, the finding should sharpen questions about dementia etiology without weakening a much simpler and better-established public health message: smoking cessation remains important for dementia prevention regardless of *APOE* genotype.
